# MRI findings for differentiating benign and malignant soft tissue tumors: a systematic review—part 2: key imaging findings

**DOI:** 10.1007/s00256-026-05155-w

**Published:** 2026-02-07

**Authors:** Muaz Wahid, Aayush Sharma, Mahad Rehman, Shyam Ramachandran, Majid Chalian, Gitanjali Bajaj, Jim S. Wu, Hillary Garner, Jonathan Samet, Shivani Ahlawat, Kambiz Motamedi, Ty Subhawong, Mark Murphey, Avneesh Chhabra

**Affiliations:** 1 Department of Radiology, UT Southwestern Medical Center, 5323 Harry Hines Blvd, Dallas, TX 75390 USA; 2 Department of Radiology, Rosalind Franklin University of Medicine and Science, North Chicago, IL USA; 3 Department of Radiology, Texas A&M University School of Medicine, College Station, TX USA; 4 Department of Radiology, University of Washington, Seattle, WA USA; 5 Department of Radiology, University of Arkansas for Medical Sciences, Little Rock, AR USA; 6 Department of Radiology, Brigham and Women’s Hospital, Harvard Medical School, Boston, MA USA; 7 Department of Radiology, Mayo Clinic, Rochester, MN USA; 8 Department of Radiology, Northwestern University Feinberg School of Medicine, Chicago, IL USA; 9 Russell H. Morgan Department of Radiology and Radiological Science, Johns Hopkins University, Baltimore, MD USA; 10 Department of Radiology and Orthopaedic Surgery, University of California Los Angeles, Los Angeles, CA USA; 11 Department of Radiology and Orthopaedic Surgery, University of Miami Miller School of Medicine, Miami, FL USA; 12 Department of Radiology, Uniformed Services University of the Health Sciences, Bethesda, MD USA; 13 American College of Radiology Institute for Radiologic Pathology (AIRP), Silver Spring, MD USA; 14 Walter Reed National Military Medical Center, Bethesda, MD USA; 15 Department of Radiology and Orthopaedic Surgery, UT Southwestern Medical Center, Dallas, TX USA; 16 Adjunct Faculty, Johns Hopkins University, Baltimore, MD USA; 17 Walton Centre for Neurosciences, Liverpool, UK

**Keywords:** Soft-tissue sarcoma, MRI, Diagnostic performance, Imaging biomarkers, Malignancy differentiation

## Abstract

**Objective:**

To synthesize magnetic resonance imaging (MRI) features and their reported diagnostic performance that differentiate benign from malignant soft-tissue tumors in alignment with the 2020 World Health Organization classification.

**Materials and methods:**

A systematic review was conducted in accordance with Preferred Reporting Items for Systematic Reviews and Meta-Analyses guidelines. PubMed, Embase, Scopus, and the Cochrane Central Register of Controlled Trials were searched through July 2024. Eligible studies reported MRI feature frequencies or diagnostic accuracy for common soft-tissue tumor subtypes. Reviews, case reports, duplicates, non-English publications, and studies outside the scope were excluded. Quality was assessed using the Quality Assessment of Diagnostic Accuracy Studies-2 (QUADAS-2).

**Results:**

Seventy-six studies met inclusion criteria. In lipomatous tumors, homogeneous fat signal and thin septa supported lipoma, whereas thick or nodular septa and enhancement favored atypical or well-differentiated liposarcoma. Myxofibrosarcoma often demonstrated an infiltrative fascial “tail.” Vascular lesions included angioleiomyoma with a reticular T2 pattern and glomus tumor with marked T2 hyperintensity and avid enhancement. In peripheral nerve sheath tumors, lower apparent diffusion coefficient values and peritumoral edema favored malignancy. Heterogeneity in imaging protocols precluded meta-analysis; results were summarized descriptively by subtype.

**Conclusion:**

Consolidated MRI patterns—such as septal morphology in lipomatous tumors, the fascial tail in myxofibrosarcoma, characteristic T2 patterns in vascular lesions, and diffusion and edema cues in nerve sheath tumors—support differentiation of benign and malignant entities, enhance reader confidence, and inform biopsy and management. Standardized prospective studies are needed to validate these thresholds and improve generalizability.

**Supplementary Information:**

The online version contains supplementary material available at 10.1007/s00256-026-05155-w.

## Introduction

Soft tissue sarcomas (STS) comprise a diverse group of tumors arising from mesenchymal tissues [1]. They are relatively rare, accounting for approximately 1% of all cancers and 2% of cancer-related deaths in the USA [2]. More than 70 histological subtypes of STS have been described, and the World Health Organization (WHO) has established a classification system to organize these entities [1].

In terms of management, tumors are routinely evaluated for evidence of malignancy before proceeding to biopsy or surgical intervention. MRI serves as the gold-standard imaging modality for extremity-related tumors, while CT is most utilized for sarcomas involving the chest and abdomen. Imaging characteristics vary across the different STS subtypes, and multiple prior studies have described the spectrum of associated findings [3]. Despite this, accurate diagnosis remains challenging, and radiologists continue to face significant pressure in distinguishing benign from malignant tumors.

Several MRI features—including septations, peritumoral edema, internal necrosis, heterogeneous enhancement, increased vascularity, diffusion restriction, and intralesional hemorrhage—have been associated with malignancy in soft-tissue tumors [4, 5]. While these imaging characteristics have been described in narrative reviews by expert radiologists, efforts to systematically consolidate these features, instantiating evidence base for their reported frequencies, and assess their diagnostic performance across tumor subtypes remain limited. Addressing this gap may improve radiologist confidence in MRI-based tumor characterization and support timely, informed clinical decision-making in patients with suspected malignant soft-tissue tumors.

In routine radiology practice, evaluation of a soft-tissue mass typically begins with identification of key MRI features, followed by progressive narrowing of the differential diagnosis in conjunction with clinical context. Initial lesion assessment commonly includes evaluation of signal homogeneity, enhancement pattern, necrosis or cystic change, peritumoral edema, septations, vascular components, diffusion restriction, and lesion margins. Additional discriminating features—such as the fascial “tail sign,” calcifications, blooming artifact related to hemosiderin, infiltrative growth, reticular T2 signal patterns, flow voids, and rim-like enhancement—may further refine diagnostic considerations. Integration of these imaging findings with lesion location, patient age, growth rate, clinical presentation, laboratory abnormalities, and complementary imaging modalities (including radiographs for mineralization, CT for calcification or hemorrhage, and ultrasound for vascularity) allows radiologists to narrow the differential diagnosis prior to biopsy or surgical intervention.

Although the present review is organized by histologic subtype according to the 2020 World Health Organization classification, the synthesized findings are intended to support a feature-first diagnostic workflow commonly used in clinical practice. The purpose of this systematic review is to consolidate key MRI features, their reported frequencies, and available diagnostic performance metrics (e.g., sensitivity and specificity) across soft-tissue tumor subtypes. By aligning these data within a standardized WHO framework, this work aims to serve as an evidence-based reference that can be readily applied in clinical interpretation and updated as new imaging-related data emerge.

## Materials and methods

### Review protocol

This review was conducted in accordance with the Preferred Reporting Items for Systematic Reviews and Meta-Analyses (PRISMA) guidelines.

### Search strategy

Institutional review board (IRB) approval was not required given the study design. A comprehensive search of PubMed, Embase, Scopus, and the Cochrane Central Register of Controlled Trials (CENTRAL) was performed from database inception through July 20, 2024, without language restrictions. Search terms combined “sarcoma,” “soft tissue sarcoma,” “MRI,” “magnetic resonance imaging,” “accuracy,” “specificity,” “sensitivity,” “grading,” and “grade.” In addition, “soft tissue sarcoma” was replaced with specific histological subtypes as defined by the 2020 WHO classification.

### Study eligibility

Studies were eligible for inclusion if they met all the following criteria:

1. Included at least 20 cases of a given common tumor type or at least five of a rare tumor.
2. Reported the frequency of sensitive or specific MRI findings.
3. Used MRI as the primary imaging modality.

Exclusion criteria were review articles, case reports, duplicates, non-English publications, studies not focused on radiologic findings, and articles outside the scope of the research aims.

### Study selection

Four independent reviewers (medical students) screened titles and abstracts, followed by a full-text review of potentially eligible studies. Discrepancies were resolved by the corresponding author. All stages of study selection, data abstraction, and interpretation were closely supervised by expert musculoskeletal radiologists as part of the American College of Radiology (ACR) soft tissue reporting and data system committee. Disagreements were resolved by consensus among the authors.

Study quality was assessed using the Quality Assessment of Diagnostic Accuracy Studies 2 (QUADAS-2) tool, which evaluates risk of bias across four domains: patient selection, index test, reference standard, and timing. Studies were required to satisfy at least seven of the 10 QUADAS-2 criteria for inclusion.

**Fig. 1:**
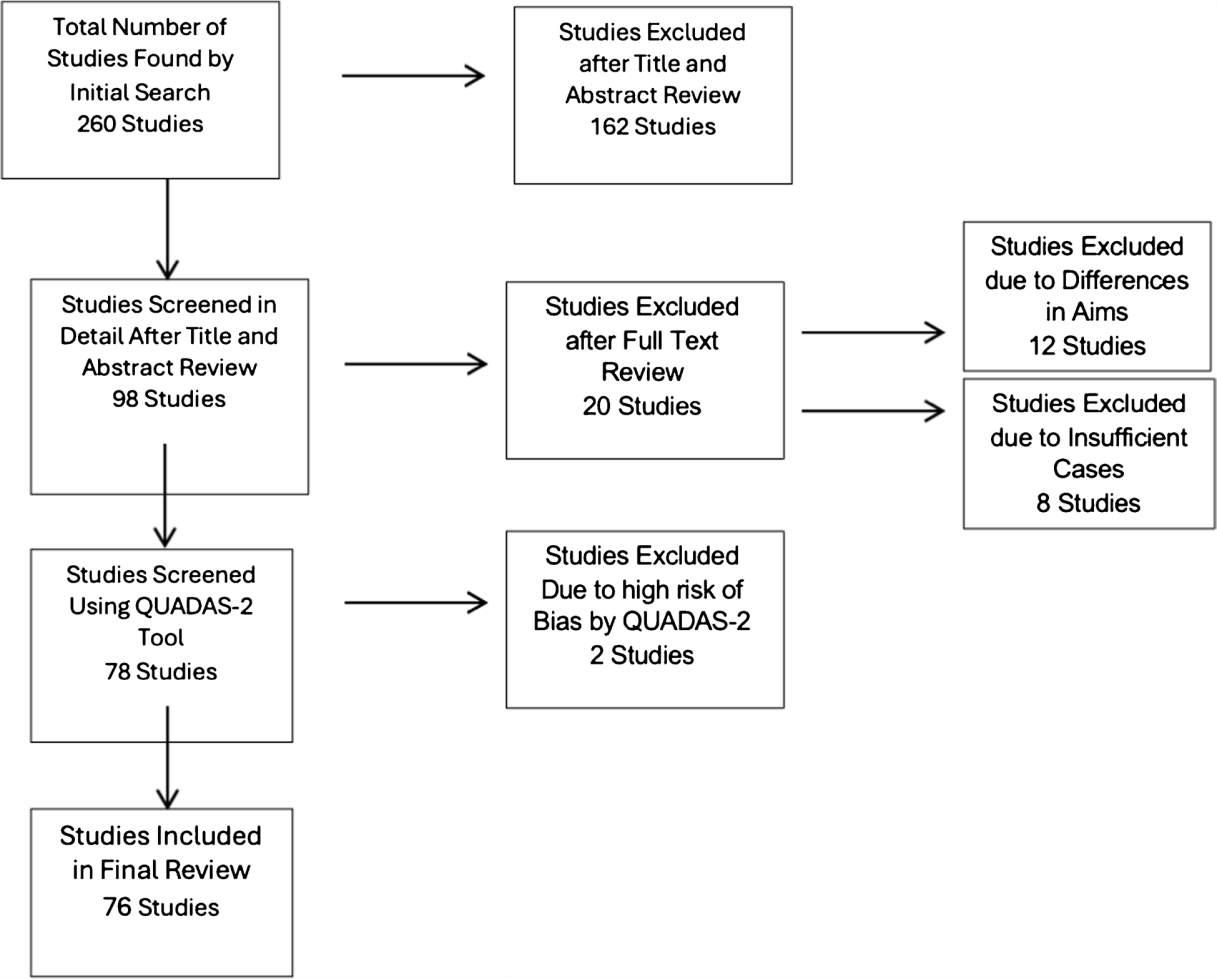
Flow diagram illustrating the systematic literature search, study selection, and inclusion process according to PRISMA guidelines

In total, 260 studies were identified. After title and abstract review, 162 were excluded as not pertinent, 12 were excluded due to differing aims, and 20 more following full-text review. An additional eight studies were excluded for insufficient case numbers, and two were excluded for high risk of bias based on QUADAS-2. Ultimately, 76 studies met all criteria and were included in the final analysis. The selection process is summarized in Fig. 1.

### Research questions

Research questions were developed collaboratively by the ACR team members to ensure clinical relevance. Categories for analysis were aligned with the WHO 2020 classification of soft tissue tumors. For example, in lipomatous tumors, one representative question was: What is the frequency of non-fatty components (e.g., septations, nodularity) in distinguishing a simple lipoma from other lesions?

All guiding questions are provided in Appendix A, and the corresponding results are summarized in Tables 1, 2, 3, and 4. Extended subtype-specific imaging frequencies are presented in Supplementary Tables 1–6. Preliminary abstraction and review were conducted by two independent medical student reviewers; however, all extracted questions, data, and interpretations were reviewed and confirmed under the supervision of senior musculoskeletal radiologists from the ACR team to ensure accuracy and methodological rigor.

**Table 1:** Lipomatous and fibroblastic/myofibroblastic tumors

Tumor type	Common finding	Number of tumors	Frequency of finding (*n*/*N*, %)	Source
Lipoma	Septa > 2 mm	8	0/8 (0%)	Coran 2017
Lipoma	Homogeneous signal	8	5/8 (62.5%)	Coran 2017
Atypical lipomatous tumor	Post-gadolinium enhancement	8	8/8 (100%)	Johnson 2018; Panzarella 2005
Spindle cell lipoma	Hyperintense septations on T1W and T2W images	37	34/37 (91.9%)	Younan 2017
Spindle cell lipoma	Contrast enhancement	26	24/26 (92.3%)	Younan 2017
Angiolipoma	Absence of vascular component	71	52/71 (73.2%)	Kransdorf 2022
Angiolipoma	Intermediate signal on fat-suppressed MRI	71	57/71 (80.3%)	Kransdorf 2022
Myxoid liposarcoma	Internal septations	28	24/28 (85.7%)	Wortman 2016
Myxoid liposarcoma	Post-gadolinium enhancement	28	26/28 (92.9%)	Wortman 2016
Nodular fasciitis	T1 isointense to skeletal muscle	29	25/29 (86.2%)	Coyle 2013
Nodular fasciitis	T2 or STIR heterogeneous signal	29	29/29 (100%)	Coyle 2013
Nodular fasciitis	Well-defined borders	29	28/29 (96.6%)	Coyle 2013
Elastofibroma dorsi	Fatty and fibrous tissue isointense to skeletal muscle on T2W images	21	21/21 (100%)	Marino 2013
Fibroma of tendon sheath	Isointense to muscle on T2W images	6	6/6 (100%)	Fox 2002
Low-grade fibromyxoid sarcoma	Hyperintense to muscle on T2W images	21	11/21 (52.4%)	Hwang 2012
Infantile fibrosarcoma	Hyperintense to muscle on T2W images	18	17/18 (94.4%)	Eleti 2022
Myxofibrosarcoma	Signal spreading along fascial planes on T2W images (tail sign)	17	14/17 (82.4%)	Kaya 2008
Fibromatosis	Hyperintense to muscle on T2W images	26	20/26 (76.9%)	Lee 2006
Plexiform fibrohistiocytic tumor	Hyperintense to muscle on fat-suppressed T2W images	5	5/5 (100%)	Ghuman 2019
Plexiform fibrohistiocytic tumor	Purely plaque-like or infiltrative morphology	8	6/8 (75.0%)	Ghuman 2019
Plexiform fibrohistiocytic tumor	Located in subcutaneous tissue	8	8/8 (100%)	Ghuman 2019
Plexiform fibrohistiocytic tumor	Isointense to muscle on non–fat-suppressed T2W images	5	4/5 (80.0%)	Ghuman 2019
Plexiform fibrohistiocytic tumor	Moderate or avid enhancement	4	4/4 (100%)	Ghuman 2019

This table summarizes reported MRI findings for adipocytic and fibroblastic/myofibroblastic tumors classified according to the 2020 World Health Organization system. For adipocytic tumors, the analysis focuses on the frequency of non-fatty components, including thick septations, nodularity, enhancement, and vascular features, which may help distinguish lesions beyond simple lipoma and characterize atypical or malignant subtypes. Additional MRI features characteristic of specific adipocytic tumor entities are also reported. For fibroblastic and myofibroblastic tumors, the table reports the frequency of signal characteristics on T2-weighted imaging (excluding fat-suppressed sequences) and other defining MRI features, such as fascial or perifascial extension, plaque-like morphology, and infiltrative growth patterns

## Results

### Lipomatous tumors

Lipomas were characterized by septations < 2 mm (100%) and homogeneity across MRI sequences in 62.5% of cases, with universal T1 hyperintensity (100%) [6]. The sensitivity and specificity for homogeneity were 89% and 62.5%, respectively, with a PPV of 93% and NPV of 50% [6]. Atypical lipomatous tumors demonstrated post-gadolinium enhancement in all cases (100%) [7, 8]. Spindle cell lipomas frequently showed hyperintense septations (91%) and contrast enhancement (86%) [9]. Gadolinium enhancement yielded 100% sensitivity and 71% specificity for atypical lipomatous tumors, with a PPV of 100% [7].

Angiolipomas were typically avascular on ultrasound (73.2%) and displayed intermediate signals in 80.5% of cases and high signal in 19.5% on fat-suppressed MRI [10]. Most angiolipomas showed moderate contrast enhancement (69.2%). Myxoid liposarcomas (LPS) demonstrated high rates of internal septations (86%) and intense gadolinium enhancement (93%) [11]. Pleomorphic liposarcomas were generally T1-weighted (T1W) isointense (93%) and T2-weighted (T2W) hyperintense (100%), with frequent necrosis/cystic change (86%), peritumoral edema (93%), rim enhancement (77%), solid/heterogeneous components (86%), and well-defined margins (71%) [11].

Studies of other liposarcoma subtypes and less common lipomatous tumors were excluded due to insufficient sample size. The complete distribution of imaging features is presented in Table 1**,** with extended features provided in Supplementary Table 1.

### Fibroblastic/myofibroblastic tumors

Nodular fasciitis lesions were typically T1W isointense to skeletal muscle (86%) and heterogeneous on T2W or short tau inversion recovery (STIR) sequences (100%), with well-defined borders and ovoid, teardrop, or bilobed morphology in 97% of cases; peritumoral edema was present in 69% [12]. Elastofibroma dorsi demonstrated fatty and fibrous tissue isointense to skeletal muscle on T2W imaging in all cases (100%) [13]. Fibromas of tendon sheaths were also consistently isointense to skeletal muscle on T2W imaging (100%) [14]. Low-grade fibromyxoid sarcomas showed variable T2 signal: 52% hyperintense and 48% hypo- to isointense relative to muscle [15].

Infantile fibrosarcomas were hyperintense to skeletal muscle in 94% of cases and enhanced with gadolinium in 83% [16]. Myxofibrosarcomas characteristically demonstrated perifascial T2 signal in 81% of cases [17]. Fibromatosis displayed T2W hyperintensity in 77% of cases [18]. Plexiform fibrohistiocytic tumors were T2W hyperintense in 80% of cases [19].

Other fibroblastic/myofibroblastic subtypes were less frequently reported. Proliferative myositis is often presented with T1 hypointensity (66.7%), T2 hyperintensity (33.3%), necrosis or cystic change (33.3%), and solid components (67%) [20]. Dermatofibrosarcoma protuberans generally appeared T1 and T2 isointense, with occasional slight T2 hyperintensity and solid components [21]. Calcifying aponeurotic fibroma consistently showed T2 hyperintensity and well-defined margins (100%) [22].

Findings across fibroblastic and myofibroblastic tumors are also detailed in Table 1, with extended frequency data in Supplementary Table 2.

### So-called fibrohistiocytic tumors/smooth muscle tumors/skeletal muscle tumors/chondro-osseous tumors

Diffuse-type tenosynovial giant cell tumors (TGCT) commonly demonstrated central hypointensity with a granular appearance on T2W imaging (83.3%) and infiltrative margins (83.3%), while lacking peripheral hypointense rims (0%)—a distinction useful for differential diagnosis, surgical planning, and disease monitoring [23]. Localized TGCTs lacked infiltrative margins (0%) and typically appeared circumscribed [23]. Low apparent diffusion coefficient (ADC) values were also characteristic, with all cases showing a mean ADC of 0.9 × 10⁻^3^ mm^2^/s (SD 0.1 × 10⁻^3^)[24]. TGCTs frequently exhibited T1 isointensity (100%), T2 isointensity (19%), calcifications (62%), and necrosis/cystic change (97%) [23].

Extraskeletal osteosarcomas were most often deep-seated (83%), with mineralization (62%), heterogeneous T2 signal (79%), and rim-like peritumoral edema (83%). Hemorrhage was observed in 37.8% of cases [25].

Rhabdomyosarcomas frequently demonstrated nodal involvement on [18]F-FDG PET-CT (80%) [26]. On MRI, they typically showed T1 isointensity (19%), T2 hyperintensity (100%), necrosis/cystic change (97%), and solid components (79%) [27]. Leiomyomas demonstrated homogeneous T2 hyperintensity and were isointense to skeletal muscle on T1-weighted imaging [28]. Dermatofibrosarcoma protuberans generally showed T1 and T2 isointensity, sometimes with slight hyperintensity and a solid component [21]. Osteosarcomas often presented with calcifications and necrosis, particularly in high-grade tumors where necrosis was present in 53% of cases [25, 29]. Solid components were also frequently observed, especially in diffuse lesions.

Studies of additional subtypes were excluded due to insufficient sample size. These results are summarized in Table 2**,** with extended MRI feature frequencies provided in Supplementary Table 3.

**Table 2:** So-called fibrohistiocytic, smooth muscle, skeletal muscle, and chondro-osseous tumors

Tumor type	Common finding	Number of tumors	Frequency of finding (*n*/*N*, %)	Source
Diffuse tenosynovial giant cell tumor	Central hypointensity/hemosiderin deposition with granular appearance on T2W images	6	5/6 (83.3%)	Jeong 2022
Diffuse tenosynovial giant cell tumor	Multinodular morphology	6	4/6 (66.7%)	Jeong 2022
Diffuse tenosynovial giant cell tumor	Infiltrative margin	6	5/6 (83.3%)	Jeong 2022
Localized tenosynovial giant cell tumor	Circumscribed margin	22	22/22 (100%)	Jeong 2022
Localized tenosynovial giant cell tumor	Single nodule	22	18/22 (81.8%)	Jeong 2022
Localized tenosynovial giant cell tumor	Septum-like hypointensity	22	20/22 (90.9%)	Jeong 2022
Localized tenosynovial giant cell tumor	Peripheral hypointense rim	22	19/22 (86.4%)	Jeong 2022
Tenosynovial giant cell tumor	Low apparent diffusion coefficient (ADC)	18	18/18 (100%)	Ashikyan 2019
Extraskeletal osteosarcoma	Peritumoral edema	47	39/47 (83.0%)	Crombé 2023
Extraskeletal osteosarcoma	Marked heterogeneity on T2W images	48	38/48 (79.2%)	Crombé 2023
Extraskeletal osteosarcoma	Marked heterogeneity on contrast-enhanced images	40	29/40 (72.5%)	Crombé 2023
Extraskeletal osteosarcoma	Well-defined or focally infiltrative margins	47	39/47 (83.0%)	Crombé 2023
Extraskeletal osteosarcoma	Mineralization	42	26/42 (61.9%)	Crombé 2023
Extraskeletal osteosarcoma	Necrosis	40	39/40 (97.5%)	Crombé 2023
Extraskeletal osteosarcoma	Rim-like peripheral enhancement	40	17/40 (42.5%)	Crombé 2023
Extraskeletal osteosarcoma	MRI fluid–fluid or hemorrhagic components	45	17/45 (37.8%)	Crombé 2023
Rhabdomyosarcoma	Nodal involvement on PET–CT	272	217/272 (79.8%)	Norman 2015

This table summarizes reported MRI findings for fibrohistiocytic, smooth muscle, skeletal muscle, and chondro-osseous tumors according to the 2020 World Health Organization classification. For tenosynovial giant cell tumors, the table reports the frequency of characteristic signal loss on T2-weighted images related to hemosiderin deposition, morphologic growth patterns, and diffusion restriction reflected by low apparent diffusion coefficient (ADC) values. For other tumor subtypes within these categories, commonly reported MRI features, including margin characteristics, heterogeneity, mineralization, necrosis, edema, and enhancement patterns, are presented

### Vascular tumors/pericytic tumors

Results are summarized in Table 4 and Supplementary Table 4. Glomus tumors consistently demonstrated T2 hyperintensity in 85.7–100% of cases, with well-defined margins and heterogeneous components observed in all instances [30–32]. Synovial hemangiomas were uniformly T1 isointense and T2 hyperintense (100%) [33].

Angioleiomyomas frequently exhibited T1 isointensity (88–100%) and T2 hyperintensity (56–100%) [34–37]. Additional features included a peripheral hypointense rim on T2W imaging (76%) and the reticular sign, also present in 76% of cases [34].

Angiosarcomas demonstrated variable MRI findings, most often with intratumoral hypointensity (86%) and heterogeneous components (71%) [38, 39]. Hemangioendotheliomas typically appeared T1 hypointense (100%) and T2 hyperintense (72.7%), with multifocal presentation (75%) and post-contrast enhancement (81.8%) [40–42].

Diagnostic features of vascular and pericytic tumors are summarized in Table 3, with expanded data presented in Supplementary Table 4.

**Table 3:** Vascular, pericytic, and peripheral nerve sheath tumors

Tumor type	Common finding	Number of tumors	Frequency of finding (*n*/*N*, %)	Source
Glomus tumor	Hyperintense to muscle on T2W images	42	36/42 (85.7%)	Al-Qattan 2005
Glomus tumor	Solid enhancement with well-defined margins	42	42/42 (100%)	Al-Qattan 2005
Synovial hemangioma	Iso- to intermediate signal on T1W and hyperintense on T2W images	4	4/4 (100%)	Greenspan 1995
Angioleiomyoma	Reticular sign on T2W images	25	19/25 (76.0%)	Edo 2021
Angioleiomyoma	Reticular sign on T2W images	80	45/80 (56.0%)	Bernard 2024
Angioleiomyoma	Isointense to muscle on T1W and T2W images	8	8/8 (100%)	Gupte 2008
Angioleiomyoma	Isointense to muscle on T1W and T2W images	18	14/18 (77.8%)	Kitagawa 2020
Angioleiomyoma	Isointense to muscle on T1W and T2W images	142	128/142 (90.1%)	Bernard 2024
Angiosarcoma	Intratumoral hypointensity	15	13/15 (86.7%)	Kawaguchi 2021
Angiosarcoma	Intratumoral hypointensity	8	7/8 (87.5%)	Isoda 2005
Angiosarcoma	Heterogeneous internal components	15	10/15 (66.7%)	Kawaguchi 2021
Angiosarcoma	Heterogeneous internal components	8	6/8 (75.0%)	Isoda 2005
Hemangioendothelioma	Hypointense on T1W images	10	10/10 (100%)	Epelboym 2019
Hemangioendothelioma	Hypointense on T1W images	2	2/2 (100%)	Errani 2012
Hemangioendothelioma	Hypointense on T1W images	25	25/25 (100%)	Hu 2018
Hemangioendothelioma	Moderate or marked enhancement	22	18/22 (81.8%)	Hu 2018
Hemangioendothelioma	Multifocal disease	29	22/29 (75.9%)	Hu 2018
Hemangioendothelioma	Hyperintense on T2W images	22	16/22 (72.7%)	Hu 2018
Peripheral nerve sheath tumor	Perilesional edema (malignant vs benign)	798	Sensitivity 60%; Specificity 94%	Wilson 2021
Peripheral nerve sheath tumor	ADC < 1.1 × 10⁻^3^ mm^2^/s (malignant vs benign)	798	Sensitivity 93%; Specificity 95%	Wilson 2021
Malignant Peripheral nerve sheath tumor	Ill-defined margins	28	22/28 (78.6%)	Jin 2023
Benign peripheral nerve sheath tumor	Well-defined margins	57	48/57 (84.2%)	Jin 2023
Malignant peripheral nerve sheath tumor	Peritumoral edema or invasion of adjacent planes	28	18/28 (64.3%)	Li 2008
Malignant peripheral nerve sheath tumor	Heterogeneous enhancement	21	11/21 (52.4%)	Jin 2023
Malignant peripheral nerve sheath tumor	Tail sign	21	6/21 (28.6%)	Chhabra 2011
Malignant peripheral nerve sheath tumor	Heterogeneous signal on T2W images	21	17/21 (81.0%)	Chhabra 2011

This table summarizes reported MRI findings for vascular, pericytic, and peripheral nerve sheath tumors classified according to the 2020 World Health Organization system. For vascular and pericytic tumors, the table reports characteristic signal intensities, enhancement patterns, morphologic features, and distribution that may aid in lesion characterization. For glomus tumors, typical signal characteristics and enhancement behavior are emphasized. For peripheral nerve sheath tumors, the table summarizes recurrent MRI features associated with benign and malignant lesions, including margin characteristics, perilesional edema, enhancement patterns, and diffusion restriction on apparent diffusion coefficient (ADC) maps. When applicable, diagnostic performance metrics (sensitivity and specificity) reported for differentiating malignant from benign tumors are presented separately from feature prevalence

### Peripheral nerve sheath tumors

Results are summarized in Table 5 and Supplementary Table 5. Perilesional edema demonstrated a 60% sensitivity and 90% specificity for distinguishing malignant from benign peripheral nerve sheath tumors (PNSTs) [43]. ADC restriction < 1.1 × 10⁻^3^ mm^2^/s showed higher diagnostic accuracy, with 93% sensitivity and 95% specificity [43].

Malignant PNSTs (MPNSTs) typically presented with ill-defined boundaries (78.6%), peritumoral edema (66%), heterogeneous T2W signal (80.9%), and the tail sign (40%) [44–48]. They also demonstrated T2W hyperintensity in 77.8–100% of cases, necrosis or cystic change in 51%, and solid components in 55–78% [44–47].

Benign PNSTs (BPNSTs) were generally well circumscribed, with well-defined margins in 84.2% of cases [44]. Schwannomas consistently showed T1 hyperintensity (100%), T2 hyperintensity (80.8%), and well-defined margins (76.9%) [45]. Perineuromas demonstrated T1 isointensity in 84% and T2 hyperintensity in 78% of cases [49]. Granular cell tumors most often appeared isointense on both T1 and T2 sequences (60%) [50].

Findings for PNSTs are also summarized in Table 3, with detailed tumor-specific imaging characteristics provided in Supplementary Table 5.

### Tumors of uncertain differentiation/undifferentiated small round cell sarcomas of bone and soft tissue

Phosphaturic mesenchymal tumors were typically T1 isointense (83%) and demonstrated solid gadolinium enhancement in most cases (93.3%) [51]. Less common findings included internal fluid–fluid levels (10.8%), intratumoral hemorrhage (10.8%), and fracture visibility on MRI/CT (42.4%) [51].

Synovial sarcomas were generally heterogeneous and infiltrative (78.4–100%), uniformly T2 hyperintense (100%), and frequently associated with peritumoral edema (73–96.1%) and solid or heterogeneous components (78–100%), especially in high-grade cases. All reported tumors demonstrated low ADC minimum values (100%) [21, 24, 52–54].

Epithelioid sarcomas were most often T1 isointense (60%) and T2 hyperintense (68.8–100%), with consistent peritumoral edema (100%) and frequent necrosis or cystic change (71–75%) [55, 56]. Alveolar soft part sarcomas were typically T2 hyperintense relative to muscle and demonstrated flow voids in 78–96% of cases [56–59].

Undifferentiated pleomorphic sarcomas were most often heterogeneous on T2W imaging (70%) [54, 60]. They demonstrated bone involvement (cortical thinning or direct extension) in 71% of cases and deep compartment invasion in 85% [53]. Ewing sarcomas were uniformly T2 hyperintense (100%) with necrosis or cystic change in 58–81.9%, solid/heterogeneous components in 76%, and well-defined margins in 67–81% [61, 62].

Perivascular epithelioid cell tumors (PEComas) frequently demonstrated necrosis (56%) and clear peritumoral borders (90%) [63]. Clear cell sarcomas showed more variable features but were often T1 hyperintense (52%) with well-defined margins (67%) [64]. Pleomorphic sarcomas were typically T2 hyperintense (59–80%) and demonstrated solid or heterogeneous components in 70% of cases [60].

Results for tumors of uncertain differentiation and undifferentiated sarcomas are summarized in Table 4, with extended subtype-specific features presented in Supplementary Table 6.

**Table 4:** Tumors of uncertain differentiation and undifferentiated small round cell sarcomas of bone and soft tissue

Tumor type	MRI finding	Number of tumors	Frequency of finding (*n*/*N*, %)	Source
Phosphaturic mesenchymal tumor	Isointense signal on T1W images	37	31/37 (83.8%)	Broski 2018
Phosphaturic mesenchymal tumor	Solid or heterogeneous gadolinium enhancement	30	28/30 (93.3%)	Broski 2018
Synovial sarcoma	Peritumoral edema	15	11/15 (73.3%)	Sedaghat 2023
Synovial sarcoma	Peritumoral edema	51	49/51 (96.1%)	Chhabra 2019
Synovial sarcoma	T2W hyperintense signal	15	15/15 (100%)	Sedaghat 2023
Synovial sarcoma	Solid or heterogeneous enhancement	15	10/15 (66.7%)	Sedaghat 2023
Synovial sarcoma	Solid or heterogeneous enhancement	15	12/15 (80.0%)	Ashikyan 2021
Synovial sarcoma	Low ADC (higher pathological grade)	15	15/15 (100%)	Ashikyan 2021
Epithelioid sarcoma	Peritumoral edema	10	10/10 (100%)	Tateishi 2002; McCarville 2019
Epithelioid sarcoma	Necrosis or cystic change	14	10/14 (71.4%)	McCarville 2019
Alveolar soft part sarcoma	Markedly hyperintense on T2W images with flow voids	18	14/18 (77.8%)	McCarville 2014
Alveolar soft part sarcoma	Markedly hyperintense on T2W images with flow voids	8	7/8 (87.5%)	Gulati 2021
Alveolar soft part sarcoma	Markedly hyperintense on T2W images with flow voids	25	24/25 (96.0%)	Crombé 2019
Undifferentiated pleomorphic sarcoma	Fascial tail sign	10	6/10 (60.0%)	Mahajan 1989
Undifferentiated pleomorphic sarcoma	Heterogeneous signal on T2W images	10	7/10 (70.0%)	Imanishi 2016
Undifferentiated small round cell sarcoma	Contiguous relationship to bone or cortical thinning	34	24/34 (70.6%)	Jones 1993
Undifferentiated small round cell sarcoma	Inhomogeneous signal on T2W images	34	28/34 (82.4%)	Jones 1993
Undifferentiated small round cell sarcoma	Deep compartment involvement	34	29/34 (85.3%)	Jones 1993
Ewing sarcoma	Hyperintense signal relative to muscle on T2W images	37	37/37 (100%)	Huh 2015
Ewing sarcoma	Necrosis or cystic change	37	30/37 (81.1%)	Huh 2015
Ewing sarcoma	Necrosis or cystic change	26	15/26 (57.7%)	Somarouthu 2014

This table summarizes reported MRI findings for tumors of uncertain differentiation and undifferentiated small round cell sarcomas involving bone and soft tissue, classified according to the 2020 World Health Organization system. The table highlights recurrent MRI features across these aggressive entities, including signal intensity on T1- and T2-weighted images, enhancement patterns, peritumoral edema, necrosis or cystic change, and characteristic morphologic findings

## Discussion

Diagnosing STS requires integrating imaging findings with the clinical presentation. The goal of this review was to consolidate reported MRI features across tumor subtypes to better assess their diagnostic value. Our synthesis highlights both the heterogeneity of imaging findings and the lack of consensus on their diagnostic utility. By collating the frequency, sensitivity, and specificity of various MRI signs across the WHO classification of STS, we sought to identify features most consistently reported across subtypes and to clarify their value in differentiating benign from malignant lesions.

**Fig. 2:**
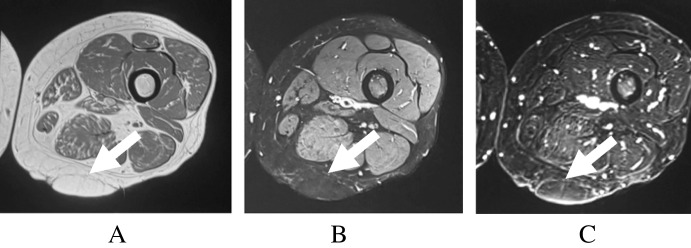
Adult patient with lipoma. Axial T1-weighted (**A**), axial fat-suppressed T2-weighted (**B**), and post-contrast T1-weighted subtraction (**C**) images demonstrate a well-defined lipomatous mass (arrows) with thin, nonenhancing septations. Diagnosis was histologically confirmed

**Fig. 3:**
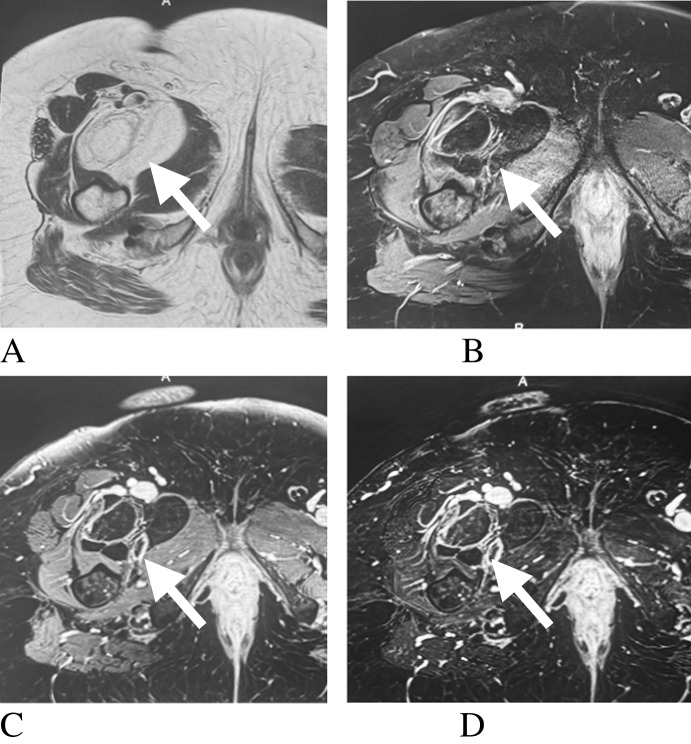
Adult patient with atypical lipomatous tumor. Axial T1-weighted (**A**), axial fat-suppressed T2-weighted (**B**), post-contrast fat-suppressed T1-weighted (**C**), and subtraction (**D**) images demonstrate a multilobulated lipomatous mass (arrows) with thick, enhancing septations. Diagnosis was histologically confirmed

Among lipomas, homogeneous signal was identified as the most diagnostically useful feature, present in 89% of tumors [6]. This corresponded with sensitivity and specificity values of 89% and 62.5%, respectively [6]. Septations greater than 2 mm were not observed in this cohort, conferring a reported specificity of 100% for malignancy, as illustrated by the thin, non-enhancing septations characteristic of lipoma (Fig. 2). Prior reviews support these distinctions, with Burt and Huang noting that thin, non-enhancing septa are typical of lipomas, whereas thick, nodular septa may indicate more aggressive pathology [65]. Consistent with this challenge, Kransdorf et al. highlighted the diagnostic difficulty in distinguishing lipomas from well-differentiated liposarcomas when irregular or enhancing septa are present, as seen in atypical lipomatous tumors with thick, enhancing septations (Fig. 3) [10]. Non-fatty components have also been associated with alternate tumor types, consistent with the findings of Brisson et al. and Berkeley et al. [66, 67].

**Fig. 4:**
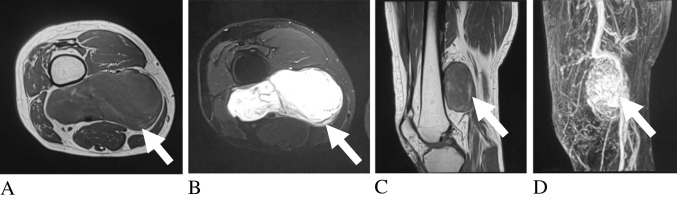
Adult patient with myxoid liposarcoma. Axial T1-weighted (**A**), axial fat-suppressed T2-weighted (**B**), sagittal T1-weighted (**C**), and post-contrast T1-weighted perfusion (**D**) images demonstrate a multilobulated soft-tissue mass (arrows) with cystic-appearing myxoid components and streaky internal fat. Diagnosis was histologically confirmed

Malignant potential was most strongly suggested by the presence of septations and contrast enhancement, particularly in spindle cell lipomas [9]. This is consistent with Jelinek et al., who observed contrast enhancement in all cases reviewed [68]. Angiolipomas, despite their nomenclature, were predominantly avascular on ultrasound, with 73% demonstrating no vascular component [10]. However, vascularity may be underestimated by ultrasound, as Kransdorf et al. reported high vascularity on CT and Hu et al. demonstrated vascular enhancement on MRI in spinal epidural angiolipomas [10, 42]. Well-differentiated liposarcomas typically display thick septations that are hyperintense on T2-weighted imaging, distinguishing them from other lipomatous tumors [69]. By contrast, myxoid liposarcomas are largely non-fatty and demonstrate marked heterogeneity, often mimicking cystic masses due to their myxoid matrix [69]. Their characteristic marked T2 hyperintensity, driven by high water content within the tumor matrix, represents a key imaging feature for differentiation, frequently producing a multilobulated mass with cystic-appearing components and internal enhancement (Fig. 4), is a key feature for differentiating benign from malignant variants, and can prevent unnecessary aggressive intervention in benign entities such as lipoblastomas [65].

In pediatric cases, lipoblastoma—a rare benign neoplasm—can mimic liposarcoma due to complex imaging features such as thick septations and non-adipose soft-tissue components. Despite this overlap, lipoblastoma is benign and occurs primarily in young children [70]. Although fat is present, septation complexity and enhancement patterns may increase recurrence risk, particularly with compartmental invasion [70]. These findings emphasize the importance of distinguishing lipoblastoma from more aggressive liposarcomas, which often necessitate more extensive surgical intervention.

For fibroblastic and myofibroblastic tumors, our results align with Haseli et al., particularly in nodular fasciitis [71]. We observed T2 hyperintensity in all cases, with peritumoral edema present in 69%, consistent with its benign nature. Nodular fasciitis also showed homogeneous T1 isointensity relative to skeletal muscle (86%) and heterogeneous T2/STIR signals (100%) [12]. Wu et al. confirmed similar T1 isointensity and T2 hyperintensity, while also identifying the fascia tail and cloud signs as significantly more frequent in nodular fasciitis than in other soft tissue lesions (*p* < 0.001 and *p* < 0.05, respectively) [72]. Morphologically, most nodular fasciitis lesions were smooth and well defined, with ovoid, teardrop, or bilobed shapes (97%). Khuu et al. reported comparable findings, including frequent T1 isointensity and oval morphology [73].

Low-grade fibromyxoid sarcomas in our analysis showed T2 hyperintensity in 62% of cases, often accompanied by solid components and necrosis, highlighting MRI’s role in assessing aggressiveness. Elastofibroma dorsi, though rare, typically contained intermixed fatty and fibrous tissue that appeared isointense to skeletal muscle on T2W imaging [13]. Deveci et al. similarly described alternating fibrous and fatty patterns [74]. Infantile fibrosarcomas were predominantly hyperintense relative to skeletal muscle on T1- and T2-weighted sequences (94%) and enhanced with gadolinium in 83% of cases [16]. Ainsworth et al. reported concordant findings, including T1 iso- to hyperintensity and T2 hyperintensity [75].

**Fig. 5:**
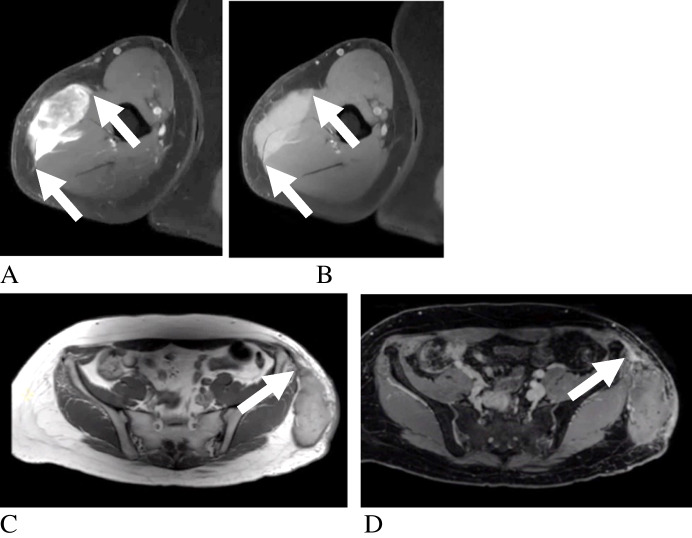
Two adult patients with myxofibrosarcoma. Axial fat-suppressed T2-weighted (**A**), axial post-contrast fat-suppressed T1-weighted (**B**), axial T2-weighted (**C**), and post-contrast fat-suppressed T1-weighted (**D**) images demonstrate heterogeneous soft-tissue masses with enhancing perifascial extensions (arrows), consistent with the fascial “tail sign.” Diagnoses were histologically confirmed

Myxofibrosarcomas frequently demonstrated perifascial T2 signal extension (82%), with the classic enhancing “tail sign” reflecting infiltrative spread along fascial planes and serving as a characteristic imaging feature (Fig. 5) [17, 76]. Fibromatosis and plexiform fibrohistiocytic tumors frequently exhibited T2 hyperintensity, consistent with Robbin et al., who also noted that fibromatoses commonly infiltrate adjacent tissues [18, 19, 77].

Our findings on tenosynovial giant cell tumors (TGCTs) were from single study by Spierenburg et al., who reported T1-weighted isointensity in all evaluated cases and identified calcifications in 62% of patients, noting that this estimate was derived from a single cohort [78]. The high necrosis rate (97%) further underscores MRI’s importance in diagnosis and monitoring. Central hypointensity with granular appearance was also observed in a majority of TGCTs (83.3%) [23]. Crim et al. demonstrated that TGCTs frequently display areas of low T1- and T2-weighted signal with blooming artifact on gradient echo, present in 86% of diffuse and 27% of nodular cases, suggesting blooming may aid in differentiating diffuse from localized disease [79]. Diffuse TGCTs were more likely to demonstrate infiltrative margins compared with localized types [23]. Low ADC values are another hallmark feature, with Ashikyan et al. reporting universally low ADC values in TGCTs [24].

Rhabdomyosarcomas demonstrated nodal involvement on PET-CT in most cases (80%), highlighting their metastatic potential [26]. While expected for malignant lesions, the high prevalence of nodal disease provides important staging information. These tumors also showed T2 hyperintensity and necrosis in 97% of cases, corroborating Allen et al.’s description of their aggressive behavior [27]. Extraskeletal osteosarcomas typically demonstrated well-defined margins (83%), mineralization (62%), peritumoral edema (83%), and hemorrhage in 38% of cases [25]. These findings are consistent with McAuley et al., who described extraskeletal osteosarcomas as well-circumscribed, T1 isointense, T2 hyperintense, and variably demonstrating hemorrhage and mineralization [80]. Leiomyosarcomas also frequently presented with T2 hyperintensity and necrosis (53%), reinforcing MRI’s role in characterizing their aggressiveness [81].

For dermatofibrosarcoma protuberans (DFSP), we observed T1 and T2 isointensity with occasional solid components, consistent with Paramythiotis et al., who emphasized DFSP’s tendency to infiltrate subcutaneous tissue, muscle, and bone [82]. These features further support MRI’s utility in evaluating local invasiveness. Similarly, our findings in osteosarcomas—marked by frequent calcification and necrosis, particularly in high-grade cases—are consistent with Wang et al., reaffirming MRI’s critical role in defining tumor extent and guiding surgical planning [83].

Glomus tumors, most commonly arising in the upper extremities, demonstrated characteristic MRI findings of T1 isointensity and T2 hyperintensity, consistent with prior reports [31, 32, 84, 85]. Al-Qattan reported MRI sensitivity of 90%, specificity of 50%, PPV of 97%, and NPV of 20% for detecting glomus tumors. Typical MRI features included T2 hyperintensity (86%), solid enhancement, and well-defined margins (100%) [30]. Patel et al. similarly reported consistent findings in hand and foot glomus tumors [86]. In addition to their classic appearance, glomus tumors may exhibit the “salt and pepper” sign on T2-weighted imaging, reflecting their hypervascular nature and aiding in the detection of smaller lesions[39].

**Fig. 6:**
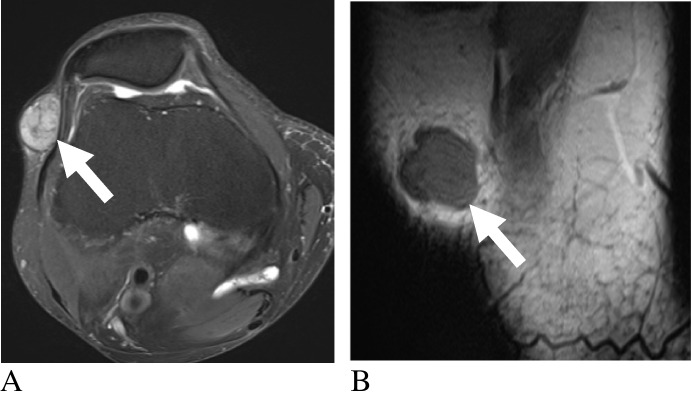
Adult patient with angioleiomyoma. Axial fat-suppressed T2-weighted (**A**) and axial T1-weighted (**B**) images demonstrate a small heterogeneous soft-tissue mass (arrows) with internal dark reticular strands. Diagnosis was histologically confirmed

For angioleiomyomas, T2W reticular signs were observed in 76% of cases (Edo 2021). Location may aid diagnosis, as Kang et al. reported that most lesions occurred in the subcutaneous fat layer (91%), were typically oval in shape (77%), and had well-circumscribed margins (100%) [87]. These tumors were almost uniformly T2W hyperintense (77–100%) [35–37]. Such findings, corroborated by Bhaludin et al., highlight the value of MRI in distinguishing benign vascular tumors from more aggressive entities such as angiosarcomas [88]. Koga et al. further emphasized the “dark reticular sign” on T2W images as a useful feature for differentiating angioleiomyomas from other vascular tumors (Fig. 6)[89].

Angiosarcomas typically exhibited intratumoral hypointensity (86%) and heterogeneous components (71%) [38, 39]. Bhaludin et al. additionally described associated hemorrhage, necrosis, and flow voids with low signal intensity in some cases [88]. Studies comparing angiosarcomas with squamous cell carcinoma of the scalp noted distinct MRI patterns, including intratumoral T2 hypointensity and mixed hyper-/hypointensity, underscoring MRI’s importance in differentiating hypervascular tumors such as glomus tumors and angiosarcomas [38].

Hemangioendotheliomas were most often T1 hypointense and T2 hyperintense with some degree of enhancement and a multifocal pattern [40–42]. Kang et al. also noted osseous hemangioendotheliomas with homogeneous post-contrast enhancement, along with increased uptake on Technetium-99m sestamibi bone scintigraphy [87]. Similarly, synovial hemangiomas typically demonstrated T1 isointensity, T2 hyperintensity, and well-defined margins, features that help distinguish them from other soft tissue masses[90].

**Fig. 7:**
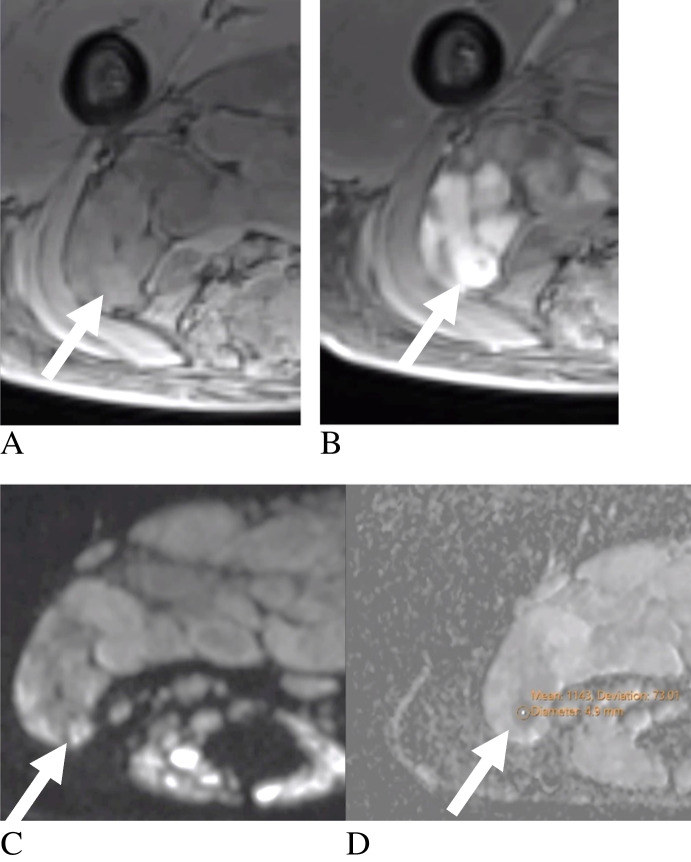
Adult patient with neurofibromatosis type 1 and malignant peripheral nerve sheath tumor. Axial fat-suppressed T1-weighted (**A**) and axial post-contrast fat-suppressed T1-weighted (**B**) images demonstrate a plexiform gluteal lesion. Diffusion-weighted imaging (**C**) and corresponding apparent diffusion coefficient map (**D**) show focal diffusion restriction (ADC = 1.1 × 10⁻^3^ mm^2^/s, arrows), consistent with malignant transformation. Diagnosis was histologically confirmed

Among peripheral nerve sheath tumors (PNSTs), perilesional edema (60% sensitivity, 94% specificity) and ADC restriction < 1.1 × 10⁻^3^ mm^2^/s (93% sensitivity, 95% specificity) were the most clinically useful indicators of malignancy, particularly when focal restriction is identified within a plexiform lesion, supporting malignant transformation in the appropriate clinical context (Fig. 7) [43]. Malignant PNSTs (MPNSTs) were more likely to exhibit poorly defined boundaries (78.6%), higher-grade vascularity (71% with Grade III), and heterogeneous enhancement (81%) [44, 48, 91]. Necrosis and the “fascicular sign” were less reliable, present in only 35.3% and 10.7% of cases, respectively [44, 45]. Both benign and malignant PNSTs were T2 hyperintense relative to muscle (95%), though peritumoral edema was more common in malignant cases (66% vs. 23%) [47]. The fascicular and tail signs showed substantial overlap between benign and malignant tumors; specifically, the tail sign was present in a minority of MPNSTs (~ 30%) and was more frequently observed in benign PNSTs, underscoring its poor specificity for malignancy [48]. These findings align with Crist et al., who described benign PNSTs as well-encapsulated with homogeneous signal [92]. In contrast, MPNSTs showed ill-defined margins, peritumoral edema, intratumoral lobulation, peripheral enhancement, and cystic degeneration [91]. In patients with neurofibromatosis 1, neurofibromas and plexiform neurofibromas often demonstrate the target sign, but malignant transformation to MPNST remains a major concern.

For phosphaturic mesenchymal tumors (PMTs), the most prevalent features were intermediate T1 signal (84%) and solid gadolinium enhancement (93%) [51]. Other studies confirmed associated T2/STIR hyperintensity [93]. Synovial sarcomas characteristically demonstrated peritumoral edema (73–96%), T2 hyperintensity (100%), solid or heterogeneous enhancement (78–100%), and uniformly low ADC values in higher-grade tumors [21, 52–54].

Epithelioid sarcomas were consistently associated with peritumoral edema (100%) and necrosis or cystic change (71–75%) [55, 56]. Yin et al. described comparable imaging in adrenal epithelioid sarcomas, with lesions appearing iso- to hyperintense on T1/T2 and enhancing homogeneously or heterogeneously [94]. Alveolar soft part sarcomas were frequently T2 hyperintense with prominent flow voids (78–96%), consistent with Spinnato et al., who noted their hyperintensity on T1/T2, peritumoral vessels, and marked vascularity [56–59, 95]. Undifferentiated pleomorphic sarcomas often exhibited a positive tail sign (60%) and heterogeneous T2 signal (70%), with case reports further supporting the diagnostic relevance of the tail sign [54, 60, 96]. Ewing sarcomas were uniformly T2 hyperintense, with necrosis or cystic change in 58–82% of cases [61, 62]. Clemente et al. confirmed that these tumors typically appear T1 isointense, T2 hyperintense, heterogeneous, and with low ADC values [97].

Overall, MRI remains the most reliable modality for characterizing soft tissue tumors, offering critical information for differentiating benign from malignant lesions. For example, PMTs often present with calcifications and well-defined margins, aiding in the identification of osseous variants [98]. By contrast, synovial sarcomas consistently demonstrate T2 hyperintensity, peritumoral edema, and rim enhancement—features essential for grading and treatment planning. The presence of solid or heterogeneous components, especially in high-grade cases, reinforces the conclusions of Riley et al. and McCarville regarding MRI’s role in both diagnosis and management [56, 98].

### Limitations

This study is limited by the heterogeneity of the topic and the available literature. Variability in imaging protocols can alter the appearance and interpretation of findings, and differences in patient selection, demographics, and comorbidities may further influence results across studies. Because of this heterogeneity, statistical consolidation was not feasible, and we elected to conduct a systematic review without meta-analysis, reporting instead the frequency of imaging findings. While this approach provides a broad overview across subtypes, it also highlights the need for larger, subtype-specific studies with standardized imaging protocols to validate the diagnostic value of these features.

## Conclusion

STS and their subtypes are challenging to diagnose on imaging, and there remains a lack of consolidated research identifying which findings have the greatest diagnostic utility. Our review sought to evaluate the diagnostic value of various imaging features across STS subtypes and to determine which are most consistently observed. The results underscore the heterogeneity of the literature and the difficulty of establishing consensus on reliable diagnostic markers. Nevertheless, several findings appear consistently across tumors and may guide diagnostic evaluation. Further studies with larger, more uniform cohorts are needed to validate these features and clarify their role in clinical practice.

The following questions were generated collaboratively with a senior musculoskeletal (MSK) radiologist to guide data abstraction. They are organized by tumor category according to the 2020 WHO classification of soft tissue tumors. Unless otherwise noted, “frequent” denotes findings present in ≥ 50% of cases and “infrequent” denotes findings present in ≤ 20% of cases.

## Supplementary Information

Below is the link to the electronic supplementary material.

ESM 1 (DOCX 32.0 KB )

## Data Availability

Not applicable. This systematic review was based entirely on data extracted from previously published studies.

## Abbreviations

STS: Soft tissue sarcomas
WHO: World Health Organization
PRISMA: Preferred Reporting Items for Systematic Reviews and Meta-Analyses
IRB: Institutional review board
QUADAS-2: Quality Assessment of Diagnostic Accuracy Studies-2
PMTs: Phosphaturic mesenchymal tumors
ADC: Apparent diffusion coefficient
STIR: Short tau inversion recovery
PET-CT: Positron emission tomography-computed tomography
