# Supplementary material for: MRI findings for differentiating benign and malignant soft tissue tumors: a systematic review—part 2: key imaging findings
Source: Skeletal Radiol. 2026 Feb 7;55(6):1279–95. doi: 10.1007/s00256-026-05155-w (PMC13139302; doi:10.1007/s00256-026-05155-w)
Supplement: Supplementary file 1 — (DOCX 32.0 KB ) [file 256_2026_5155_MOESM1_ESM.docx]

**SUPPLEMENTARY MATERIALS**

**Supplementary Table 1. MRI Characteristics of Lipomatous Tumors**

| Tumor Type | Study | Number of Tumors | T1 Signal (hypo / iso / hyper, %) | T2 Signal (hypo / iso / hyper, %) | Fat Component (%) | Enhancement / Solid Component (%) | Aggressive Features† (%) | Well-Defined Margins (%) |
| --- | --- | --- | --- | --- | --- | --- | --- | --- |
| Lipoma | Coran 2017 | 8 | – | – | – | 37.5 (3/8) | – | – |
| Atypical Lipomatous Tumor | Johnson 2018 | – | – | – | – | – | – | 100 |
| Spindle Cell Lipoma | Younan 2017 | 74 | – | Hyper 86 (64/74) | 57 (>75%); 38 (<75%) | Septal 35; Solid 61 | – | – |
| Lipoblastoma | Rajput 2022 | 12 | Hyper 42 (5/12) | Hyper 100 (12/12) | 54–100 | 42 (5/12) | – | 100 |
| Lipoblastoma | Jelinek 1993 | 48 | Hyper 25 (12/48) | – | 54 (26/48) | 85 (41/48) | Hem 6; Calc 8 | 83 (40/48) |
| Lipoblastoma | Degnan 2021 | 56 | – | – | – | 59 (33/56) | Nec 13 | 100 |
| Angiolipoma | Kransdorf 2022 | 778 lesions | – | Hyper 19.5 (8/41) | 85 (661/778) | – | – | 69 (18/26) |
| Hibernoma | Lee 2006 | 8 | Hypo 50; Hyper 50 | Hypo 50; Hyper 50 | – | 100 (8/8) | – | 100 (8/8) |
| Atypical Lipomatous Tumor | Wortman 2016 | 10 | Iso 20; Hyper 80 | Hyper 100 | 90 (9/10) | 70 (7/10) | – | 100 (10/10) |
| Myxoid Liposarcoma | Wortman 2016 | 28 | Iso 100 | Hyper 100 | 36 (10/28) | 93 (26/28) | Nec 32; Edema 32 | 86 (24/28) |
| Dedifferentiated Liposarcoma | Wortman 2016 | 4 | Iso 75; Hyper 25 | Hyper 75; Iso 25 | 100 (4/4) | 100 (4/4) | Nec 75; Edema 100 | 25 (1/4) |
| Myxoid Liposarcoma | El Ouni 2010 | 12 | Iso 100 | Hyper 100 | 75 (9/12) | 100 (12/12) | Nec 17 | 100 (12/12) |
| Pleomorphic Liposarcoma | Wortman 2016 | 14 | Iso 93; Hyper 7 | Hyper 100 | 29 (4/14) | 77 (10/13) | Nec 86; Edema 93 | 71 (10/14) |

† Aggressive features include hemorrhage (hem), calcification (calc), necrosis (nec)/cystic change, or peritumoral edema when reported.

**Supplementary Table 2. MRI Characteristics of Fibroblastic and Myofibroblastic Tumors**

| Tumor Type | Study | N | T1 Signal (hypo/ iso/ hyper, %) | T2 Signal (hypo/ iso/ hyper, %) | Fat Component (%) | Enhancement /Solid Component (%) | Aggressive Features† (%) | Well-Defined Margins (%) |
| --- | --- | --- | --- | --- | --- | --- | --- | --- |
| Nodular Fasciitis | Coyle 2013 | 29 | Iso 86; Hyper 14 | Hyper 100 | – | Rim 76; Solid 100 | Nec 41; Edema 69 | 97 |
| Proliferative Myositis | Gan 2019 | 3 | Hypo 66.7; Iso 33.3; Hyper 33.3 | Hyper 100 | – | – | Hem 33.3 | – |
| Ischemic Fasciitis | Ilaslan 2005 | 3 | Iso 100 | Hyper 100 | – | – | Nec 100 | 100 |
| Fibroma of Tendon Sheath | Fox 2003 | 6 | Hypo 83; Iso 83; Hyper 17 | Hypo 50; Iso 50; Hyper 50 | – | Rim 67; Solid 83 | – | 100 |
| Elastofibroma Dorsi | Marino 2013 | 14 | – | – | 100 | Rim 100 | – | – |
| Elastofibroma Dorsi | Naylor 1995 | 21 | Hypo 90; Iso 90 | Hypo 90; Iso 90 | – | Rim 90 | Calc 5; Edema 10 | 0 |
| Elastofibroma Dorsi | Tsubakimoto 2017 | 73 | Iso 100 | Iso 100 | 77 | – | – | – |
| Low-Grade Fibromyxoid Sarcoma | Hwang 2012 | 22 | Hypo 41; Iso 68 | Hyper 59 | – | Solid 62 | Nec 62 | 62 |
| Infantile Fibrosarcoma | Eleti 2022 | 18 | Hypo 11; Iso 72; Hyper 17 | Hyper 94 | – | Solid 83 | Hem 33; Calc 17; Nec 33 | 78 |
| Myxofibrosarcoma | Kaya 2008 | 21 | Hypo 100 | Hyper 100 | – | – | Hem 9 | 81 |
| Fibromyxoid Sarcoma | Evans 2011 | 33 | Hypo 100; Iso 100 | Variable* | – | – | – | – |
| Fibromatosis | Lee 2006 | 29 | Iso 83; Hyper 17 | Hypo 31; Hyper 77 | – | Solid 65 | – | 54 |

† Aggressive features include hemorrhage (hem), calcification (calc), necrosis (nec)/cystic change, or peritumoral edema when reported.
* Reported qualitatively (“few” / “most”) in the original study.

**Supplementary Table 3. MRI Characteristics of So-Called Fibrohistiocytic, Smooth Muscle, Skeletal Muscle, and Chondro-Osseous Tumors**

| Tumor Type | Study | N | T1 Signal (hypo / iso / hyper, %) | T2 Signal (hypo / iso / hyper, %) | Enhancement / Solid or Nodular Component (%) | Aggressive Features† (%) | Well-Defined Margins (%) |
| --- | --- | --- | --- | --- | --- | --- | --- |
| Localized Tenosynovial Giant Cell Tumor | Jeong 2022 | 22 | Iso 83 | Hyper 77 | Nodules 81.8; Rim hypointense 86.4 | – | 100 |
| Diffuse Tenosynovial Giant Cell Tumor | Jeong 2022 | 6 | Iso 83.3 | Hyper 83.3 | Nodules 66.7; Hem 83.3 | Rim hypointense 0 | 16.7 |
| Rhabdomyosarcoma | Allen 2007 | 26 | Iso 81; Hyper 19 | Hyper 100 | – | Nec 65 | 35 |
| Osteosarcoma | Crombe 2023; Wang 2024; Roller 2018 | 48, 11, 16 | Hypo 15–100; Iso 65–100; Hyper 21 | Hypo 8; Hyper 85–100; Iso 6 | Solid 100 | Hem 38; Calc 64; Nec 56–97; Edema 69–83 | 30 |
| Leiomyomas | Szolomayer 2018 | 8 | Iso 100 | Hyper 100 | – | – | 100 |

† Aggressive features include hemorrhage (hem), calcification (calc), necrosis (nec)/cystic change, or peritumoral edema when reported.

## **Supplementary Table 4. MRI Characteristics of Vascular and Pericytic Tumors**

| Tumor Type | Study | N | ****T1 Signal**** (hypo / iso / hyper, %) | ****T2 Signal**** (hypo / iso / hyper, %) | ****Enhancement / Solid Component (%)**** | ****Aggressive Features† (%)**** | ****Well-Defined Margins (%)**** |
| --- | --- | --- | --- | --- | --- | --- | --- |
| Glomus Tumor | Al-Qattan 2005; Drape 1995; Theumann 2002 | 42, 28, 24 | Hypo 12.5; Iso 58; Hyper 29.1 | Hypo 8.3; Hyper 67–85.7; Iso 25 | Solid 80.8 | – | 62–100 |
| Synovial Hemangioma | Greenspan 1995 | 4 | Iso 100 | Hyper 100 | – | – | 25 |
| Angioleiomyoma | Edo 2021; Gupte 2008; Kitagawa 2020; Bernard 2024 | 25, 8, 18, 82 | Hypo 8; Iso 88–100; Hyper 4 | Hypo 44; Hyper 56–100 | Solid 80 | Hem 4–5.6; Nec 6–17 | – |
| Angiosarcoma | Kawaguchi 2021; Isoda 2005 | 15, 8 | Iso 100; Hyper 29 | Hypo 86; Hyper 71–100 | Solid 43 | – | 71 |
| Hemangioendothelioma | Epelboym 2019; Errani 2012; Hu 2018 | 10, 2, 22 | Iso 68; Hyper 31.8 | Hyper 72 | Solid 77 | Hem 9; Nec 18 | – |

† Aggressive features include hemorrhage (hem), calcification (calc), necrosis (nec)/cystic change, or peritumoral edema when reported.

**Supplementary Table 5. MRI Characteristics of Peripheral Nerve Sheath Tumors**

| Tumor Type | Study | N | T1 Signal (hypo / iso / hyper, %) | T2 Signal (hypo / iso / hyper, %) | Enhancement / Solid Component (%) | Aggressive Features† (%) | Well-Defined Margins (%) |
| --- | --- | --- | --- | --- | --- | --- | --- |
| Schwannoma | Li 2008 | 26 | Hyper 100 | Hypo 19.2; Hyper 80.8 | Solid 38.5 | – | 76.9 |
| Neurofibroma | Lim 2004 | 122 | – | – | – | – | 23–32 |
| Perineurioma | Mauermann 2009 | 32 | Hyper 84 | Hyper 78; Iso 8 | – | – | – |
| Granular Cell Tumor | Blacksin 2005 | 5 | Iso 60; Hyper 40 | Hypo 20; Iso 60; Hyper 20 | Solid 40 | – | 40 |
| Malignant Peripheral Nerve Sheath Tumor | Jin 2023; Li 2008; Wasa 2009; Demehri 2014; Chhabra 2011 | 28, 9, 41, 9, 21 | Iso 0; Hyper 70–100 | Hypo 22.2; Hyper 77.8–100 | – | Calc 28.5; Nec 4.8; Edema 52 | 47.6 |

† Aggressive features include hemorrhage (hem), calcification (calc), necrosis (nec)/cystic change, or peritumoral edema when reported.

## **Supplementary Table 6. MRI Characteristics of Tumors of Uncertain Differentiation and Undifferentiated Small Round Cell Sarcomas**

| Tumor Type | Study | N | ****T1 Signal**** (hypo / iso / hyper, %) | ****T2 Signal**** (hypo / iso / hyper, %) | ****Enhancement / Solid Component (%)**** | ****Aggressive Features† (%)**** | ****Well-Defined Margins (%)**** |
| --- | --- | --- | --- | --- | --- | --- | --- |
| Phosphaturic Mesenchymal Tumor | Broski 2018 | 37 | Iso 83.8 | Hypo 11.1; Hyper 38.9; Iso 25 | Solid 23.3 | Hem 10.8; Nec 8.1; Edema 6.7 | 75 (osseous) |
| Perivascular Epithelioid Cell Tumors | Izubuchi 2023 | 9 | – | – | – | Nec 56 | 90 |
| Synovial Sarcoma | Sedaghat 2023; Ashikyan 2021; Chhabra 2019; Jones 1993; Mahajan 1989; Tordjman 2023 | 15–98 | Hypo 5.9–20; Iso 25.5–80; Hyper 10–68.6 | Hyper 80–100; Iso 0–35 | Solid 78.4–100 | Hem 33–44; Calc 20; Nec 29–41; Edema 73–96 | 80 |
| Epithelioid Sarcoma | Tateishi 2002; McCarville 2019 | 16, 10 | Iso 60; Hyper 25–30 | Hyper 68.8–100 | Solid 37.5–50 | Hem 40–75; Calc 12.5; Nec 71–75 | 40 |
| Alveolar Soft Part Sarcoma | McCarville 2014; Gulati 2021; Lorigan 1989; Crombe 2019 | 3–25 | Iso 17–20; Hyper 78–96 | Hyper 78–96 | Solid 62.5; Flow voids 69–96 | Nec 31–42; Edema 9 | – |
| Clear Cell Sarcoma | De Beuckeleer 2000 | 21 | Hypo 33; Iso 15; Hyper 52 | Hypo 17; Iso 33; Hyper 50 | Solid 50 | Hem 12; Calc 21; Nec 33; Edema 20 | 67 |
| Extraskeletal Myxoid Chondrosarcoma | Kapoor 2014; Tateishi 2006 | 7, 19 | Iso 80–100; Hyper 26.3 | Hyper 100 | Solid 32 | Nec 23–26 | 74 |
| Pleomorphic Sarcoma | Imanishi 2016 | 10 | – | Hypo 20; Hyper 59–80 | – | – | 70 |
| Ewing Sarcoma | Huh 2015; Somarouthu 2014 | 37, 19 | Hypo 26–51.4; Iso 48.6–74 | Hyper 100 | Solid 75.6 | Nec 58–71.9 | 66.7–81 |

† Aggressive features include hemorrhage (hem), calcification (calc), necrosis (nec)/cystic change, or peritumoral edema when reported.

**Appendix A. Structured Questions for Data Abstraction**

The following questions were generated collaboratively with a senior musculoskeletal (MSK) radiologist to guide data abstraction. They are organized by tumor category according to the 2020 WHO classification of soft tissue tumors. Unless otherwise noted, “frequent” denotes findings present in ≥50% of cases and “infrequent” denotes findings present in ≤20% of cases.

| Tumor Category (WHO 2020) | Guiding Questions |
| --- | --- |
| Lipomatous Tumors | - Frequency of non-fatty components (septations, nodularity, vascularity) in distinguishing lipoma vs non-lipoma. - Frequency of solid nodules in identifying high‑grade liposarcoma. - Other characteristic MRI signs (e.g., homogeneity, septal enhancement, rim enhancement). - Which signs occur ≥50% (frequent) or ≤20% (infrequent)? |
| Fibroblastic / Myofibroblastic Tumors | - Frequency of T2‑weighted hypointensity or hyperintensity (excluding fat‑suppressed sequences). - Presence of specific signs (e.g., fascial tail sign, perifascial spread, infiltrative morphology). - Which MRI features are observed ≥50% or ≤20%? |
| So‑Called Fibrohistiocytic / Smooth Muscle / Skeletal Muscle / Chondro‑Osseous Tumors | - Is central T2 hypointensity (hemosiderin deposition, granular appearance) a consistent sign of TGCT? - Is low apparent diffusion coefficient (ADC) a reliable biomarker for TGCT? - Subtype‑distinguishing features (e.g., multinodularity, infiltrative margins, calcification, necrosis, peri‑tumoral edema). - Which findings occur ≥50% or ≤20%? |
| Vascular / Pericytic Tumors | - Does proximity to vessels or adjacent vascularity predict tumor type? - Characteristic MRI signs (e.g., reticular sign, rim enhancement, flow voids). - Does anatomic location (e.g., subcutaneous tissue, nail bed, deep soft tissue) aid subtype diagnosis (e.g., glomus tumor)? - Which findings occur ≥50% or ≤20%? |
| Peripheral Nerve Sheath Tumors (PNSTs) | - Does perilesional edema differentiate malignant from benign PNSTs? - Does ADC restriction (< 1.1 × 10⁻³ mm²/s) help classify PNSTs? - Specific signs (e.g., tail sign, fascicular sign, well‑defined margins, enhancement patterns). - Which MRI findings are consistently present (≥50%) or absent (≤20%)? |
| Tumors of Uncertain Differentiation / Undifferentiated Small Round Cell Sarcomas | - Characteristic MRI signs (e.g., T2 hyperintensity, peritumoral edema, necrosis, cortical thinning, deep compartment involvement). - Distinctive features by subtype (e.g., synovial sarcoma, epithelioid sarcoma, alveolar soft part sarcoma, Ewing sarcoma). - Which findings occur ≥50% or ≤20%? |
